# Isolation, identification, structural elucidation and bioactivity of Heneicos-1-ene from *Coriandrum sativum* L. foliage

**DOI:** 10.1038/s41598-018-35836-z

**Published:** 2018-11-27

**Authors:** Siddharth Priyadarshi, Nanishankar V. Harohally, C. Roopavathi, Madeneni Madhava Naidu

**Affiliations:** 1 Academy of Scientific and Innovative Research (AcSIR), CSIR-CFTRI Campus, Mysuru, Karnataka India; 2 Department of Spices and Flavour Sciences, CSIR-CFTRI, Mysuru, Karnataka India; 3 Microbiology & Fermentation Technology, CSIR-CFTRI, Mysuru, Karnataka India

## Abstract

Coriander foliage is a distinctive spice employed on a daily basis in curry and other Indian traditional food preparations mainly for the unique flavour attributes and health benefits. Radical scavenging activity has been demonstrated previously for coriander foliage. However, specific molecules responsible were not identified. A new molecule was isolated via chromatographic technique, and its structure was established by employing multinuclei and multidimensional NMR and HRMS techniques. The identified molecule Heneicos-1-ene was also screened for radical scavenging activity and antimicrobial activity, wherein it displayed radical scavenging activity of 89.6 ± 0.62% at 200 ppm, and also exhibited substantial antimicrobial activity against *E. coli* and *Salmonella typhi*.

## Introduction

*Coriandrum sativum* L. belongs to the family *Apiaceae*. It exists in two categories; small-fruited *C. sativum* L. var. *microcarpum DC* and large-fruited *C. sativum* L. var. *vulgare alef*^1^. The leaves, fruits and stems have a pleasant aromatic aroma hence used as a flavouring substance. The entire parts of the plant are suitable for eating, but mainly the dried seeds and fresh green leaves are used in various culinary preparation. The whole coriander plant is used in chutneys preparation, and the leaves, in particular, are used for flavouring many curries, sauces and soups. Coriander oleoresin and oils are used primarily in the bakery, beverages, chewing gums, condiments, curry mix, sweets, tobacco goods, seasonings for sausages and other meat products^1^. The various phytonutrients present in coriander endow with a range of pharmacological benefits such as stomachic, stimulant, lipolytic, insecticidal, hypolipidemic, hypoglycemic, fungicidal, diuretic diaphoretic cytotoxic, carminative, antispasmodic, antimutagenic, antibacterial, and aflatoxin protective potential^2^.

Previously our group has subjected the coriander foliage for radical scavenging activity^3^ and found that ethanol extract had indeed exhibited commendable activity when compared with 95% of butylated hydroxyanisole (BHA). We envisioned to identify the molecule responsible for antioxidant activity. With this background, we set out to extract and identify the antioxidant molecule via chromatographic separation. We present here, isolation, and identification of Heneicos-1-ene from the coriander foliage as well as its radical scavenging and antimicrobial activities.

## Reagents and Chemicals

Chemicals and solvents used for analysis were of analytical grade and procured from Merck, Mumbai, India.

## Materials and Methods

Fresh coriander (*Coriandrum sativum* L.) foliage of *Microcarpum DC* (M.DC) variety was obtained from the local producer (Mysuru, Karnataka, India) on the day of the experiment. The collected green foliage was subjected to a pretreatment; roots and extraneous foreign materials were removed. The foliages were washed with fresh water to remove any dust and other adhering particles and then were spread on filter paper at room temperature to remove excess moisture. Washed foliages (1.5 Kg) were dried for 3.5 hours using low-temperature low humidity (LTLH) dryer maintained at 52.6 ± 2.0 °C air temperature and 28.0 ± 3.0% relative humidity. The dried foliage was powdered in a mixer grinder (Panasonic, Model MX-AC400, Haryana, India) to 52 mesh size. The powder was placed in an airtight aluminium pouch and stored in the dark at room temperature for further analysis.

### Isolation, identification and structural elucidation

#### Separation using thin layer chromatography

Isolation of the molecule was carried out by thin layer chromatography (TLC) from the extract prepared from dried coriander foliage using 70% ethanol as solvent. TLC aluminium plates (20 × 20 cm) pre-coated with silica gel 60 F_254_ were obtained from Merck (Darmstadt, Germany). Developing systems of different composition were tried and optimized to get good separation. Sample solutions were applied to the plates using a capillary tube. All TLC plates were developed using an initial mobile phase of methanol: chloroform (30:70 v/v) followed by twice elution with methanol: chloroform (10:90 v/v). Linear ascending plate development was performed in a suitable chromatographic tank previously saturated for one hour with the developing mobile phase, and the scanning was carried out at 270 nm. The bands obtained were carefully excised from the plate and extracted using 2 mL chloroform. All the chloroform extracts were then pooled; dried in a rotary evaporator (Heidolph, Germany) at 30–35 °C under reduced pressure (40 millibars) and redissolved in a known volume of chloroform.

#### Spectroscopic measurement

The extracts were subjected to high-resolution mass spectrometry (HRMS) and nuclear magnetic resonance (NMR) spectroscopy for structural elucidation and identification of the molecule. Solution NMR spectral data recorded on a Bruker Avance instrument having the ^1^H frequency of 500 MHz and ^13^C frequency of 125 MHz. NMR spectral analysis was accomplished by employing the combination of experiments consisting of 1D NMR techniques ^1^H, ^13^C{^1^H}, and 2D NMR techniques involving HSQC (heteronuclear single quantum coherence), HMBC (heteronuclear multiple bond correlation), and TOCSY (total correlation spectroscopy). Mass spectra were recorded in SCIEX 5600 Q-TOF instrument.

### Determination of free radical scavenging activity by DPPH

The free radical scavenging activity of isolated Heneicos-1-ene from coriander foliage was measured by the 2, 2-diphenyl-1-picrylhydrazyl (DPPH) method^4^. The various concentrations (50, 100, and 200 ppm) of extracts were taken in different test tubes. The volume was made up to 1 mL by using respective solvent. Then 4 mL of 0.1 mM methanol solution of DPPH was added to each test tube and vortexed. The tubes were stored for 20 minutes in the dark at 21 ± 2 °C temperature. Control was prepared as above without Heneicos-1-ene, and the respective solvent was used for the baseline correction. The variation in the absorbance of the samples was measured at 517 nm. BHA was used as a standard antioxidant for comparison. The average values of the three analyses were taken. The free radical scavenging activity was expressed as the inhibition percentage and was calculated using the following formula-

$${\rm{Radical}}\,{\rm{scavenging}}\,{\rm{activity}}\,( \% )=\frac{({\rm{A}}{}_{{\rm{control}}}-{{\rm{A}}}_{{\rm{sample}}})}{{\rm{A}}{}_{{\rm{control}}}}\times 100$$

where, A_sample_ and A_control_ are the equilibrium absorbance of the test sample and the control, respectively.

### Determination of antimicrobial activity

#### Disc diffusion assay

The antibacterial activity assays were carried out using disk diffusion method^5^ and spot on lawn method using *Staphylococcus aureus*, *Escherichia coli*, *Micrococcus luteus*, *Salmonella typhi* and *Listeria monocytogens* as test organisms. The Muller-Hinton agar plates inoculated with equal to 0.5 McFarland turbidity of pathogen bacteria by sterile swab. Standard blank disk (6.0 mm diameter) was put on a plate and inoculated with 50 µL of the isolated component (10 mg). All Petri plates were incubated for 24 hours at 37 °C. Zones of inhibition measured after 24 hours. The control discs contained DMSO (−ve) and Ampicillin (+ve).

#### Spot on lawn assay

The assay was carried out on Muller Hinton agar. Muller Hinton agar plates were inoculated with different pathogen bacteria matched to 0.5 McFarland turbidity of by sterile swab. 10 µL of the isolated component was spotted on the media and incubated at 37 °C.

#### Minimum inhibitory concentration (MIC) and Minimum bactericidal concentration (MBC)

The antimicrobial activity of the isolated compound was determined by adjusting the turbidity of the bacterial pathogen to match a McFarland No. 0.5 standard (~10^7^ CFU/mL) and used for the assay^6^. A stock solution of purified compound (2 mg/mL) was prepared and serially diluted in a Muller Hinton broth (100 µL) medium dispensed in a 96-well cell culture plate to obtain a concentration of 100, 50, 25, 12.5, 6.25, 3.125, 1.56 and 0.781 µg/mL. Each well was further added with 100 µL of inoculum. The control wells were maintained with and without samples. Ampicillin (10 µg/mL) was used as reference antibiotic. The plates were sealed using parafilm and incubated for 24 hours at 37 °C. The turbidity of the broth was measured at 600 nm using titer plate reader (Thermo Scientific, USA). The minimum concentration of compound at which it inhibited the visible growth of pathogenic bacteria was taken as minimum inhibitory concentration (MIC). MBC is the lowest concentration of compound preventing the visual growth of the bacterial cultures and reduced the pathogen by ≥99.9%, irrespective of counts of survivors at higher antibiotic concentrations.

## Results and Discussion

### Structural elucidation

**Figure 1:**
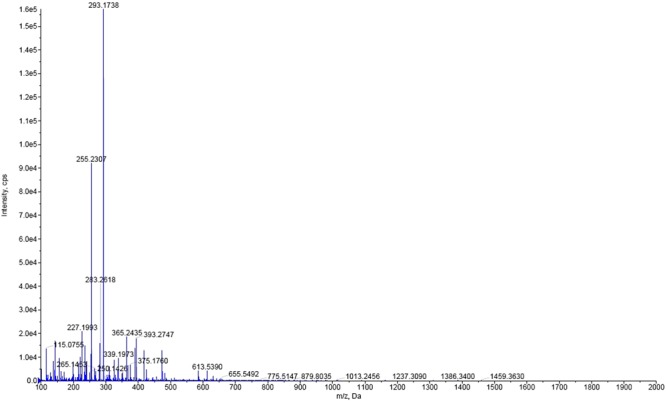
HRMS ESI-negative mode spectrum of Heneicos-1-ene.

Structural elucidation was carried out by employing high-resolution mass spectrometry (HRMS) and ^1^H, ^13^C, HMBC, HSQC and TOCSY NMR experiments. HRMS ESI-positive mode spectrum did not reveal the occurrence of molecular ion peak. However, it exhibited a peak pattern corresponding to 302.3049 assigned to (M-CH_3_ + Na)^+^, whereas the dominant peak pattern{please refer Figure I under supplementary information} centered at 274.2739 was assigned to (M-C_3_H_7_ + Na)^+^. To observe the molecular ion peak, we switched to HRMS ESI-negative mode spectrum (Fig. 1) which indicated a strong peak pattern centered at 293.1738 corresponding to (M-H)^−1^ wherein, M = C_21_H_42_.

**Figure 2:**
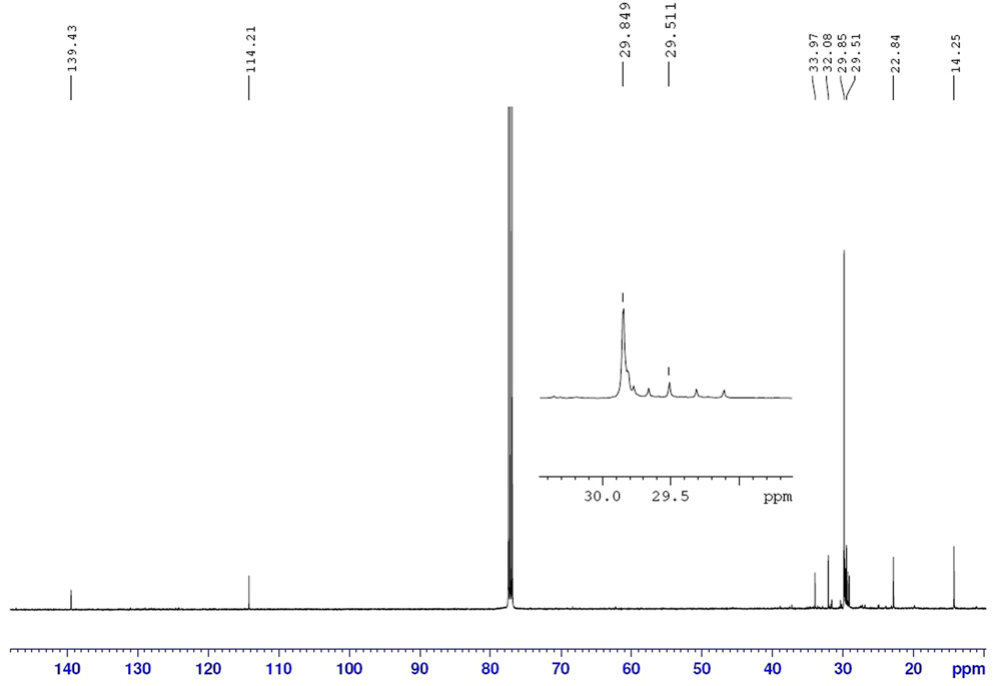
^13^C NMR spectrum of Heneicos-1-ene.

^1^H NMR revealed {please refer Figure II under supplementary information} the occurrence of CH_3_, CH_2_, CH and magnetically not equivalent protons (indicated via splitting pattern) of CH_2_ groups were observed at chemical shift of 0.88, 1.25, 5.82 and 4.96 ppm, respectively. An additional CH_2_ was also seen at chemical shift 2.04 ppm. The integral of 1.25 ppm peak indicated the occurrence of several CH_2_ peaks. On the other hand, ^13^C NMR displayed (Fig. 2) peaks corresponding to chemical shift of 14.25, 22.84, 29.51, 29.84, 32.08, 33.97, 114.21 and 139.42 ppm. The occurrence of peaks at chemical shift of 114.21 and 139. 42 ppm confirmed the presence of the C=C double bond and indicated the chemical shift values typical of the terminal alkene. HSQC spectrum {please refer Figure III under supplementary information} facilitated the assignment of ^13^C NMR for methyl and methylene groups.

**Figure 3:**
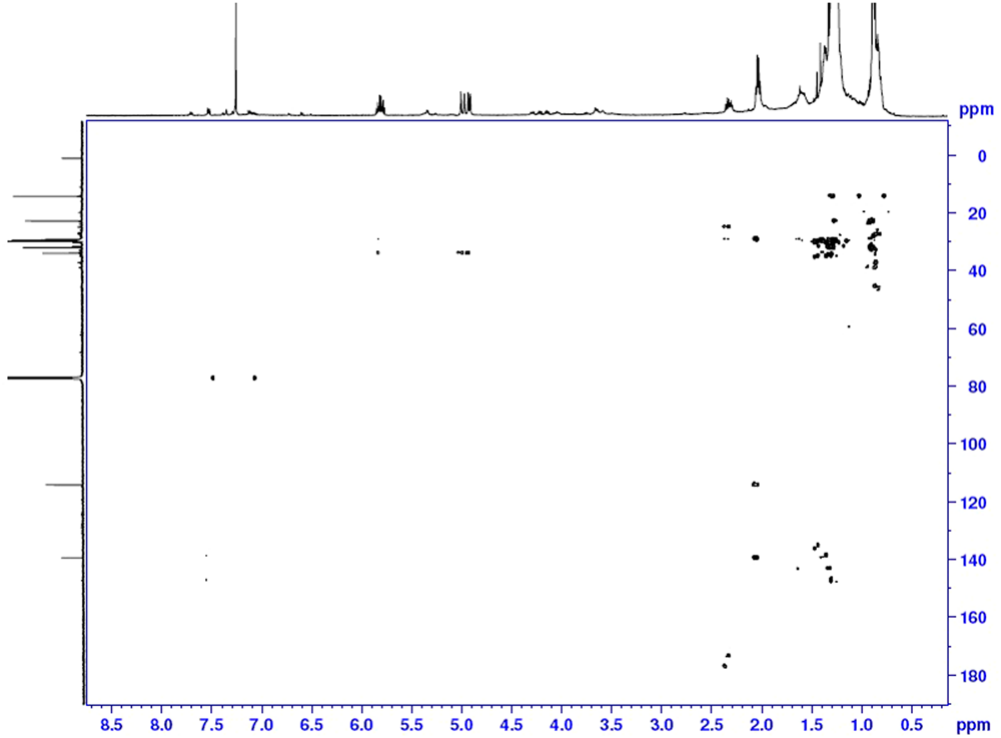
The HMBC spectrum of Heneicos-1-ene.

**Figure 4:**
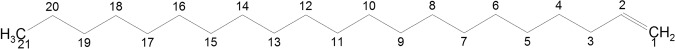
Structure of molecule identified: Heneicos-1-ene.

Further, HBMC cross peaks (Fig. 3) were observed for the peak of ^1^H chemical shift corresponding to 0.88 to 22.84 ppm and 32.94 ppm (^13^C NMR) confirming the methyl correlation with adjacent CH_2_ groups. Also, ^1^H peak corresponding to chemical shift at 2.04 ppm displayed HMBC cross peaks with ^13^C NMR peaks of chemical shift 114.21 and 139.42 ppm confirming the correlation of CH_2_ group adjacent with CH and terminal CH_2_ groups. This was also substantiated by cross peaks of ^1^H NMR peaks having the chemical shift of 4.96 and 5.82 ppm with 33.97 ppm of ^13^C NMR. Further, TOCSY experiments {please refer Fig. IV under supplementary information} established the correlations via observation of various peaks relating to spin systems and confirmed the molecule to be Heneicos-1-ene (Fig. 4). Heneicos-1-ene is synthetically made by decarbonylation reaction of docosanoic acid (CH_3_(CH_2_)_20_COOH) with Vaska’s complex utilizing potassium iodide and acetic anhydride as additive^7^. It has also been made using enzymatic oxidative decarboxylation^8^. This molecule forms the part of essential oil of blue-coloured hybrid tea rose flowers^9^.

### NMR Data

^1^H NMR(CDCl_3_): 0.88 (t, J = 6.9 Hz, 3 H, C(21)H_3_), 1.25(m, C(3)H- C(19)H_2_), 2.04(m, 2 H,C(20)H_2_), 4.96(m, 2 H,C(1)H_2_), 5.82(m,1 H,C(2)H).

^13^C NMR(CDCl_3_): 14.25(C(21)), 22.54(C(20)), 32.08(C(19)), 29.51(C(18)), 29.84(C(4)-C(17), 33.97(C(3)), 114.21(C(2)), 139.42(C(1)).

### Radical Scavenging Activity

**Figure 5:**
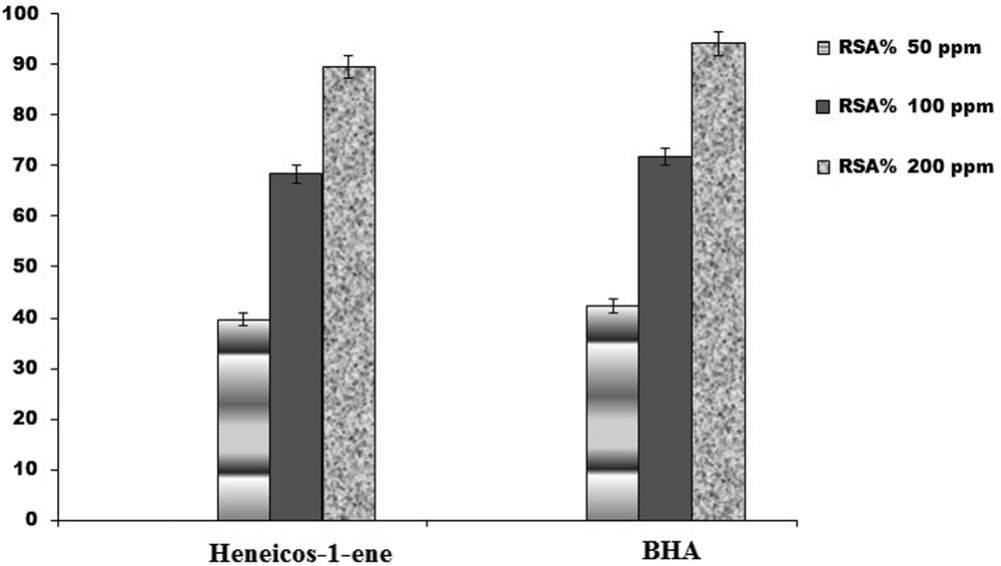
Radical scavenging activity of Heneicos-1-ene and BHA at various concentrations.

The isolated non-volatile compound Heneicos-1-ene from coriander foliage and BHA at various concentrations (50, 100 and 200 ppm) were prepared by dissolving in the chloroform. As can be observed from Fig. 5; the maximum radical scavenging activity of 89.6 ± 0.62% was attained at 200 ppm, and activity decreased with decreasing concentration of compound when compared with 95% of BHA. Heneicos-1-ene exhibited comparable radical scavenging activity when compared with the artificial antioxidant BHA.

### *In-vitro* antibacterial activity

**Figure 6:**
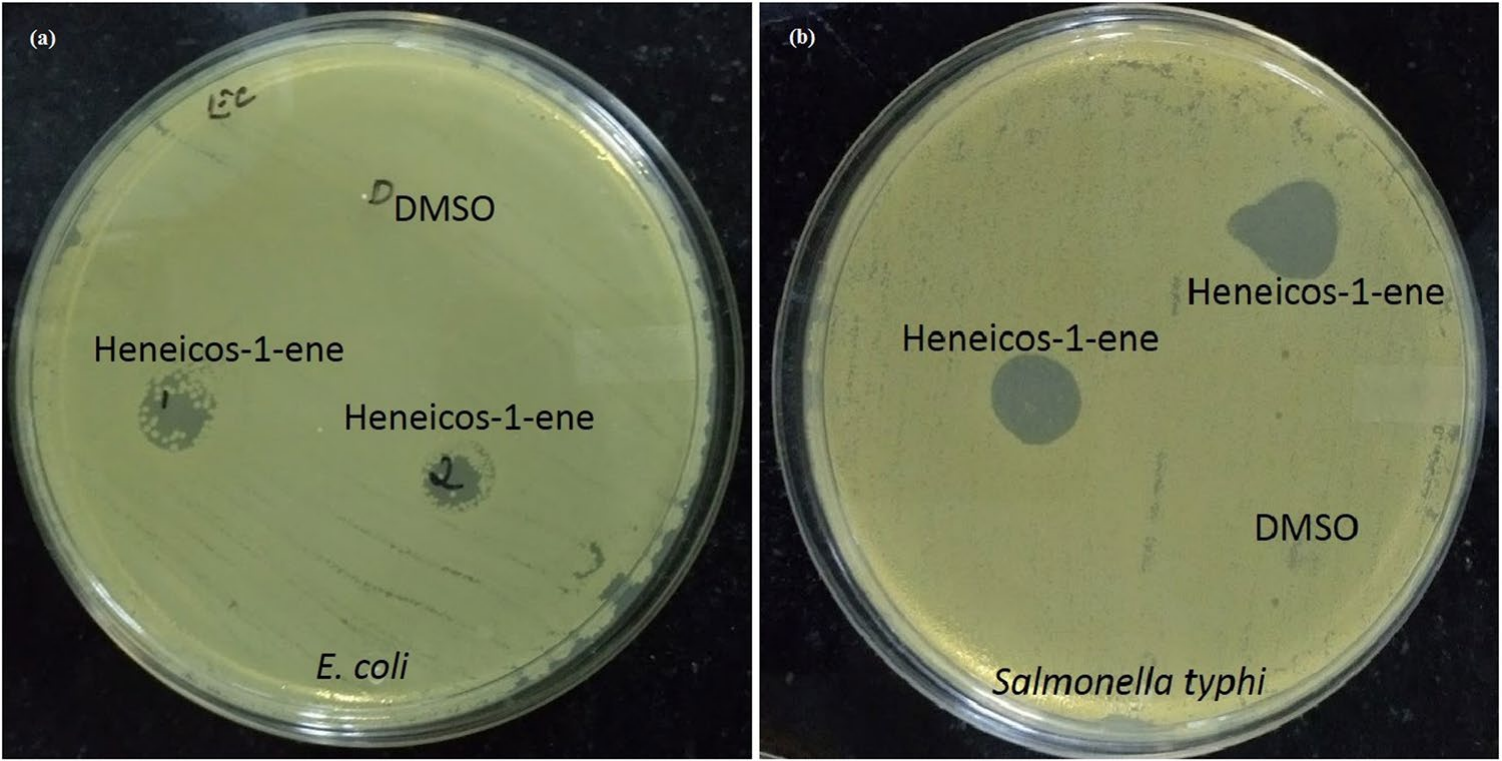
Antibacterial activity of Heneicos-1-ene against (**a**) *E. coli* and (**b**) *Salmonella typhi*.

Antibacterial activity of both aqueous crude extract and pure compound (Heneicos-1-ene) dissolved in chloroform were tested *in-vitro* by using disc diffusion method, spot on the lawn and minimal inhibition concentration method. Heneicos-1-ene has inhibited the growth of *E. coli* and *Salmonella typhi* at the concentration of 10 mg with a zone of inhibition 8.9 ± 0.079 mm and 11 ± 0.025 mm, respectively (Table 1). The zone of inhibition of the Heneicos-1-ene was more prominent against *Salmonella typhi* than *E. coli* (Fig. 6). There was no significant antibacterial activity observed against the other three tested bacterial pathogens such as *Staphylococcus aureus*, *Listeria monocytogens* and *Micrococcus luteus*. However, the aqueous crude extract was not effective in inhibiting the growth of any pathogens used in the study.

**Table 1:** The zone of inhibition of Heneicos-1-ene and antibiotic ampicillin tested against bacterial pathogens.

Bacterial pathogens	Chloroform extract (10 mg/mL)	Ampicillin§ (25 µg/mL)
*E. coli*	8.9 ± 0.079 mm	23.3 ± 0.9 mm
*Salmonella typhi*	11 ± 0.025 mm	22.8 ± 0.7 mm

^§^6 mm disc as standard

With regards to the antibacterial activity, the Minimal Inhibition Concentration (MIC) values of Heneicos-1-ene against *E. coli* and *Salmonella typhi* of the compound were confirmed using broth dilution microtiter system and expressed data as the mean ± standard deviation (SD, n = 3). The antimicrobial activity of Heneicos-1-ene was found in the range of 25 to 50 µg/mL and the minimal bacterial concentration (MBC) was in the range of 50–100 µg/mL (Table 2) for *E. coli* and *Salmonella typhi*. MBC were generally within a two-fold dilution of the MICs. Since the Heneicos-1-ene did not show any inhibition zone against other pathogens, the MIC and MBC were not determined.

**Table 2:** The MIC and MBC values of Heneicos-1-ene and antibiotics tested against bacterial pathogens.

Sample	Test Microorganisms MIC/MBC (µg/mL)	
	*E. coli*	*Salmonella typhi*
Ampicillin	0.312/0.625	1.25/0.625
Chloroform extract	50/100	25/50

Several bioactive peptides, volatile compounds^10^ and essential oils^11,12^ isolated from coriander have been accounted for a broad range of antimicrobial activity. However, our study for the first time characterizes and confirms the antimicrobial activity of Heneicos-1-ene against *E. coli* and *Salmonella typhi* and possesses a promising role in maintaining the shelf life of the food products by preventing spoilage.

## Conclusion

We isolated Heniocose-1-ene from a natural food source coriander foliage for the first time. Heniocose-1-ene exhibited excellent radical scavenging and antimicrobial activity.

## Electronic supplementary material

Supplementary information

## Data Availability

Complete data of the manuscript is available with Corresponding author.
