# Supplementary material for: Isolation, identification, structural elucidation and bioactivity of Heneicos-1-ene from Coriandrum sativum L. foliage
Source: Sci Rep. 2018 Nov 27;8:17414. doi: 10.1038/s41598-018-35836-z (PMC6258703; doi:10.1038/s41598-018-35836-z)
Supplement: Supplementary file 1 — Supplementary information [file 41598_2018_35836_MOESM1_ESM.pdf]

Isolation, identification, structural elucidation and bioactivity of Heneicos-1-ene from *Coriandrum sativum* L. foliage

Siddharth Priyadarshi[1, 2], Nanishankar V. Harohally[1, 2], Roopavathi C.[3], Madeneni Madhava Naidu[1, 2,*]

Figure No.	Figure Title	Page No.
Figure-I	HRMS ESI-positive mode spectrum of Heneicos-1-ene	2
Figure-II	^{1}H NMR spectrum of Heneicos-1-ene	3
Figure-III	HSQC spectrum of Heneicos-1-ene	4
Figure-IV	TOCSY spectrum of Heneicos-1-ene	5

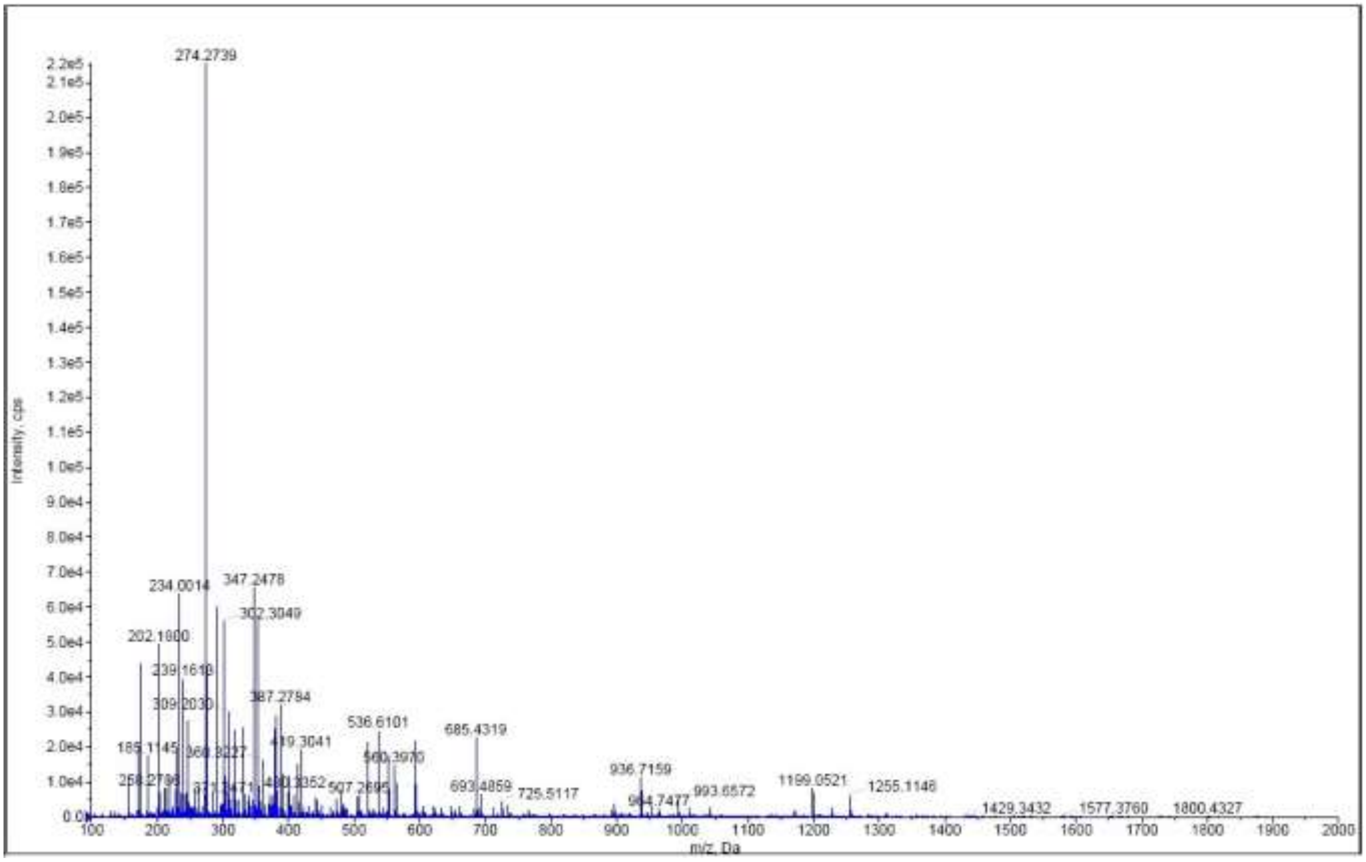

Figure-I (Madeneni Madhava Naidu) HRMS ESI-positive mode spectrum of Heneicos-1-ene

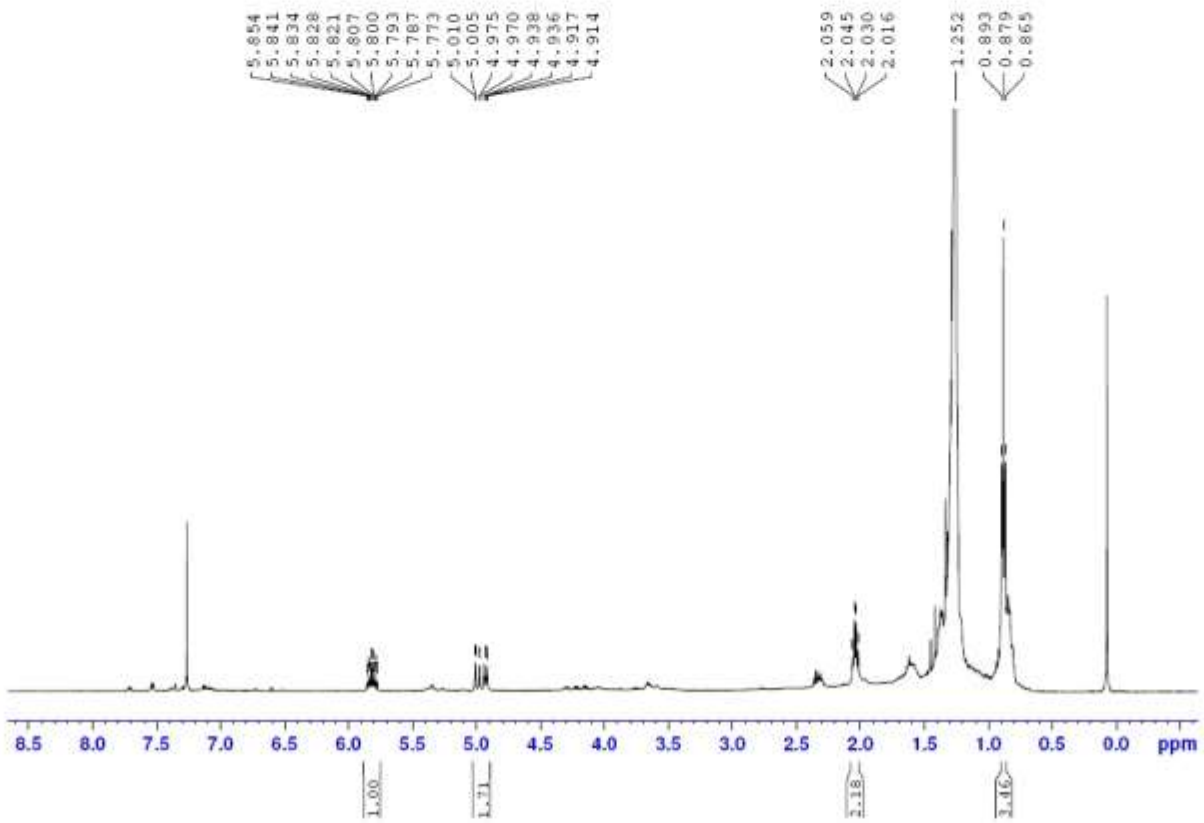

Figure-II (Madeneni Madhava Naidu) ^{1}H NMR spectrum of Heneicos-1-ene

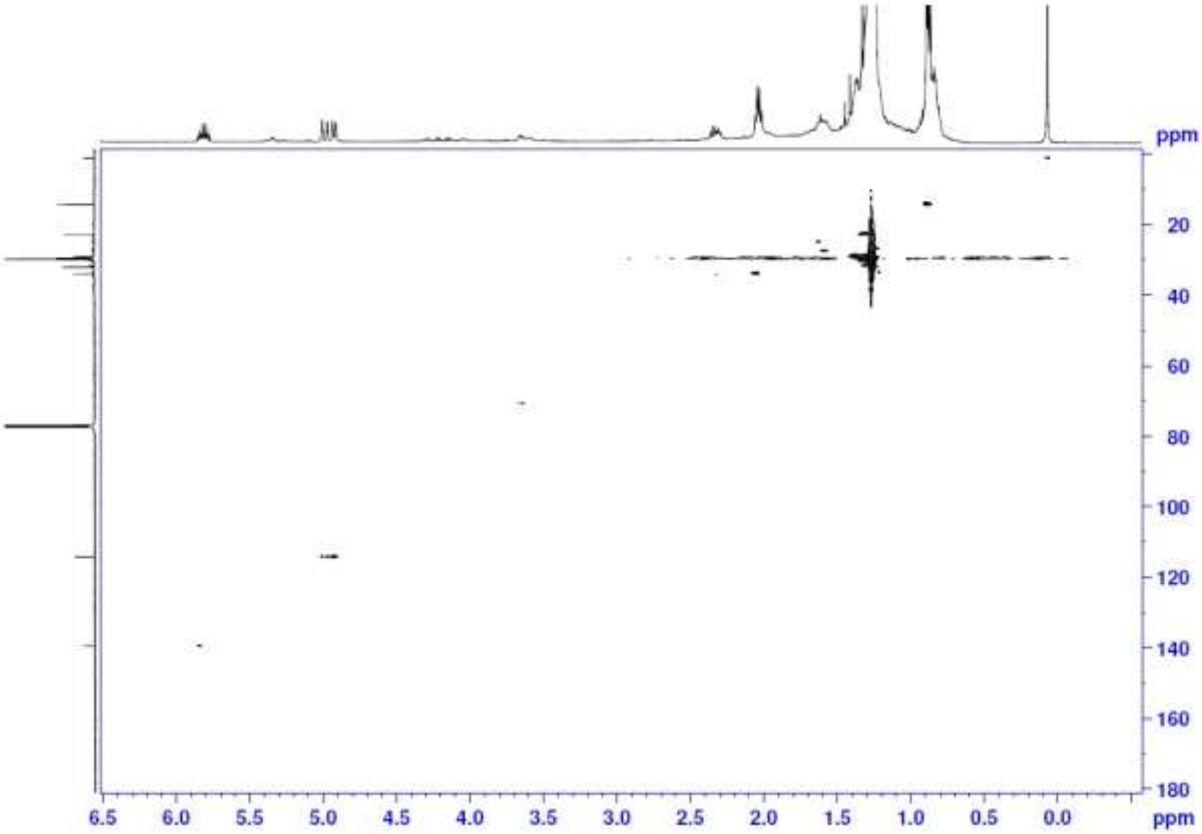

Figure-III (Madeneni Madhava Naidu) HSQC spectrum of Heneicos-1-ene

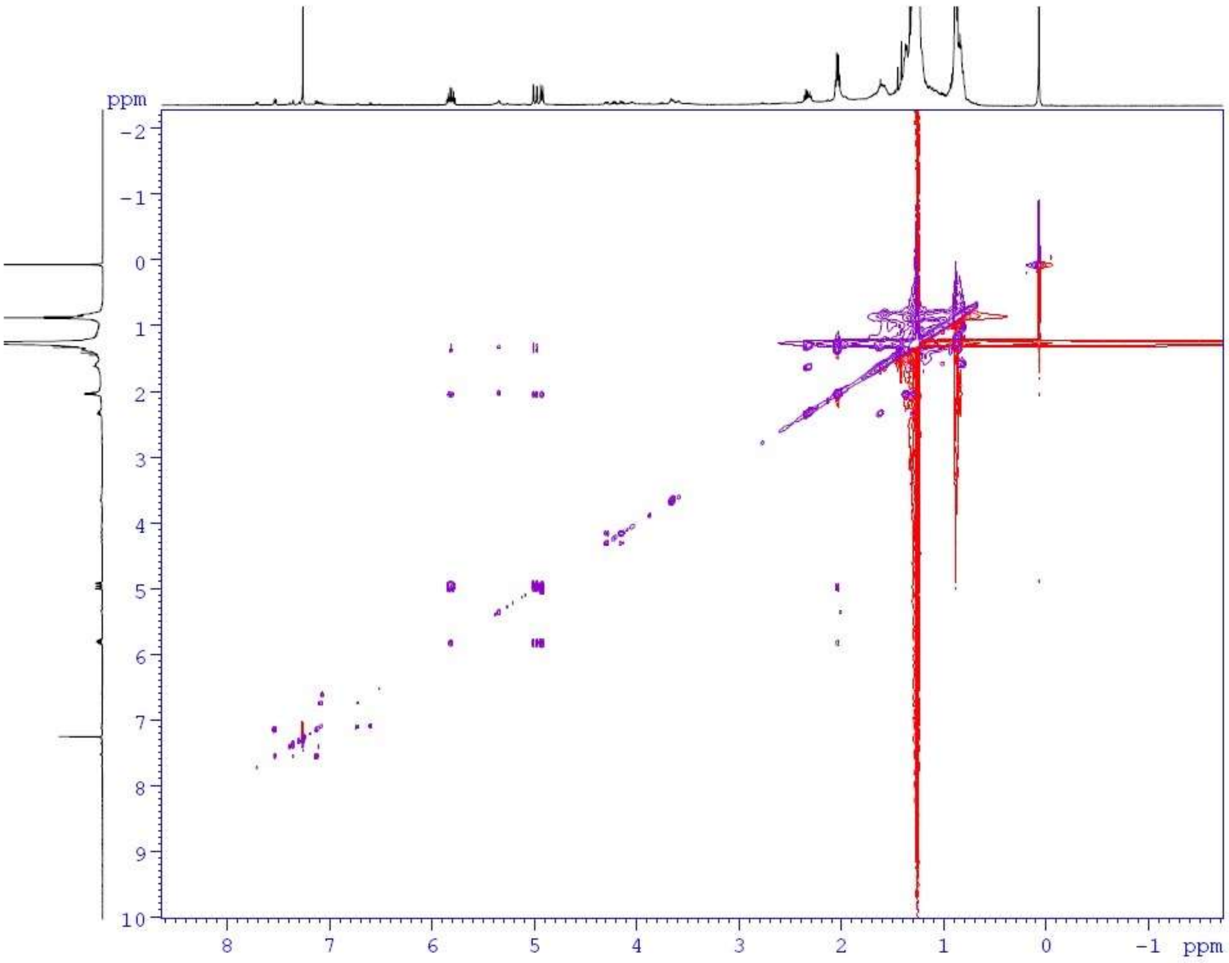

Figure-IV (Madeneni Madhava Naidu) TOCSY spectrum of Heneicos-1-ene
